# Sustained AAV9-mediated expression of a non-self protein in the CNS of non-human primates after immunomodulation

**DOI:** 10.1371/journal.pone.0198154

**Published:** 2018-06-06

**Authors:** Arlene I. Ramsingh, Steven J. Gray, Andrew Reilly, Michael Koday, Debbie Bratt, Merika Treants Koday, Robert Murnane, Jeremy Smedley, Yuhui Hu, Anne Messer, Deborah Heydenburg Fuller

**Affiliations:** 1 NYVAX Inc., Miami, Florida, United States of America; 2 Gene Therapy Center, University of North Carolina at Chapel Hill, Chapel Hill, North Carolina, United States of America; 3 Department of Pediatrics, University of Texas Southwestern Medical Center, Dallas, Texas, United States of America; 4 Wadsworth Center, New York State Department of Health, Albany, New York, United States of America; 5 Washington National Primate Research Center, Seattle, Washington, United States of America; 6 Department of Microbiology, University of Washington, Seattle, Washington, United States of America; 7 Neural Stem Cell Institute, Rensselaer, New York, United States of America; University of Kansas Medical Center, UNITED STATES

## Abstract

A critical issue in transgene delivery studies is immune reactivity to the transgene- encoded protein and its impact on sustained gene expression. Here, we test the hypothesis that immunomodulation by rapamycin can decrease immune reactivity after intrathecal AAV9 delivery of a transgene (GFP) in non-human primates, resulting in sustained GFP expression in the CNS. We show that rapamycin treatment clearly reduced the overall immunogenicity of the AAV9/GFP vector by lowering GFP- and AAV9-specific antibody responses, and decreasing T cell responses including cytokine and cytolytic effector responses. Spinal cord GFP protein expression was sustained for twelve weeks, with no toxicity. Immune correlates of robust transgene expression include negligible GFP-specific CD4 and CD8 T cell responses, absence of GFP-specific IFN-γ producing T cells, and absence of GFP-specific cytotoxic T cells, which support the hypothesis that decreased T cell reactivity results in sustained transgene expression. These data strongly support the use of modest doses of rapamycin to modulate immune responses for intrathecal gene therapies, and potentially a much wider range of viral vector-based therapeutics.

## Introduction

Giant axonal neuropathy (GAN, OMIM# 256850) is a rare, autosomal recessive pediatric neurodegenerative disease, characterized by progressive motor, sensory, and CNS axonal neuropathy[1]. Loss of the function of the encoded protein, gigaxonin, leads to dysregulation and accumulation of intermediate filaments (IFs), including structural neurofilaments (NF-H, NF-M, and NF-L), vimentin, peripherin, alpha-internexin, desmin, keratin and vimentin[2, 3]. The giant axonal swellings increasingly disrupt critical neuronal functions in a wide range of neurons, with death commonly occurring in the second and third decades of affected individuals. GAN knockout mice develop IF aggregates, but this phenotype can be reversed using gene therapy that delivers low levels of intact gigaxonin[4]. This gene replacement approach was also successful in human motor neurons derived from patient induced pluripotent stem cells, with no adverse effects on the cultured neurons[5]. Based on these studies, a Phase I gene therapy clinical trial in children with mild to mid-stage GAN was started at NIH/NINDS (https://clinicaltrials.gov/ct2/show/NCT02362438). The trial uses intrathecal (IT) delivery of a scAAV9/JeT-GAN viral vector, with a deliberately weak promoter to more closely mimic the naturally low levels of the gigaxonin protein. Inclusion was initially restricted to patients where protein was mutated (missense), but expressed, due to concerns about the immunogenicity of a fully novel transgene in null individuals.

Although gene delivery within the CNS (including IT) is thought to provide some level of immune privilege, evidence suggests that with CNS delivery the induction of immune responses to the transgene are more likely to be delayed than eliminated. In fact, the limitations of the privilege were first noted in 1948 where immune responses were induced following transplantation of skin homografts to the brain [6]. Furthermore the blood-brain barrier (BBB) is compromised with neurodegenerations [7].

To address the issue of immunogenicity in CNS gene therapy, we tested the capacity of the known immunomodulator rapamycin (an mTOR inhibitor) to suppress immune responses and enhance expression of a model non-self transgene (GFP) delivered using the same AAV9 vector and low-level promoter that is being used in an ongoing GAN clinical trial.

## Results

### Intrathecal delivery of AAV9 with a GFP transgene with a low promoter does not induce adverse clinical effects

Cynomolgus macaques (N = 4 per group) were randomized into two treatment groups. For both groups, AAV9 vectors (scAAV9/JeT-GFP, total of 8.5 x 10^12^ vg) were administered intrathecally (IT). Each group received either AAV9/GFP only or AAV9/GFP with rapamycin administered orally daily for one week prior to and continuing for two weeks post-AAV9 (0.5 mg/kg rapamycin on day 1 and then 0.2 mg/kg each day thereafter). This dose induces immune dampening rather than full immune suppression in the rapamycin group. Blood, CSF and lymph nodes were collected at several time points throughout the study to measure immune responses. Twelve weeks after AAV9 inoculation, all animals were euthanized and necropsied to measure immune responses and evaluate GFP expression in tissues (S1 Fig). Macaques in both groups remained active, exhibited normal weight gain, normal motor function and no adverse clinical events for the 13 week duration of the study. In addition, analysis of CBC values and blood chemistries showed no significant changes during the entire follow-up period of twelve weeks following the IT AAV9 injections (S1 Table). At necropsy, histopathology showed no evidence of inflammation (immune cell infiltrates including neutrophils, macrophages, plasma cells), no changes in brain tissues and minimal to mild effects (rare activated glial cells, necrosis or fibrosis; and possible low levels of demyelination, axonal loss, and/or neuronal degeneration) in the spinal cord and dorsal ganglia.

### Antibody responses to GFP and the AAV9 capsid are dampened with rapamycin

**Fig 1:**
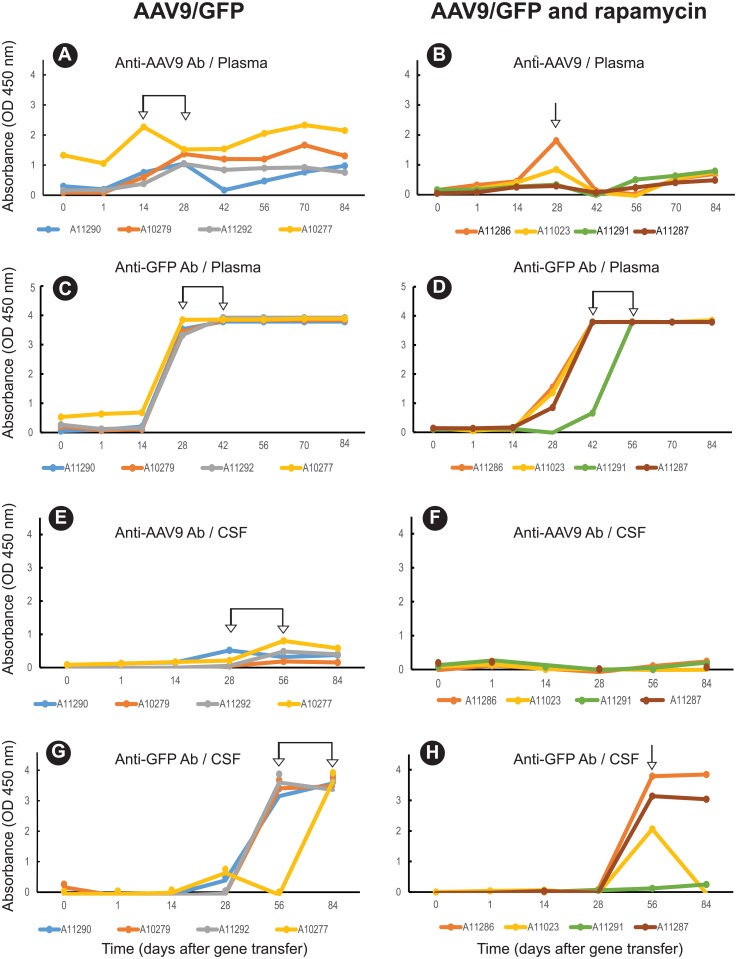
Kinetics and magnitude of anti-GFP and anti-AAV9 antibody (Ab) responses altered after treatment with rapamycin. Antibodies in plasma (A-D) and CSF (E-H) (1/250 dilution) were measured by ELISA in two treatment groups; AAV9/GFP (controls) and AAV9/GFP + rapamycin. Arrows indicate the time points or timeframes of peak Ab response.

GFP and AAV9 capsid-specific IgG antibody levels in macaque serum and cerebral spinal fluid (CSF) were assessed by enzyme-linked immunosorbent assay (ELISA) (Fig 1). Logistic regression analysis was used to model the antibody data. Three estimated parameters, Background (value at Time = Delay), Slope (rate of increase), and Delay (time before change is observed), were obtained by non-linear least squares and used to test differences in changes over time among treatment groups. Non-linear least squares also produces a Residual Sum of Squares (RSS) as a measure of how well the estimated model fits the responses. In this analysis, RSS values ranged from 0.05 to 0.1, which suggest that the estimated model is a good fit of the data. The observed Maximum response, not an estimate, was also tested (and permits utilization of the entire antibody dilution series). Of the two antigens (AAV9 and GFP), GFP was more immunogenic, inducing stronger antibody responses than the AAV9 capsid in both the plasma (Fig 1A and 1B vs. Fig 1C and 1D, P<0.001) and CSF (Fig 1E and 1F vs. Fig 1G and 1H, P <0.01). Although GFP induced higher antibody responses, the capsid antigen induced peak antibody responses in the plasma earlier when compared to GFP (Fig 1A and 1B vs. Fig 1C and 1D), a result that may be due to delayed GFP transgene expression relative to the AAV9 capsid. There was also a compartmentalized difference in the antibody response as both AAV9 capsid and GFP antibody were higher (P<0.01) and occurred earlier in plasma (Fig 1A–1D) than in the CSF (Fig 1E–1H).

**Fig 2:**
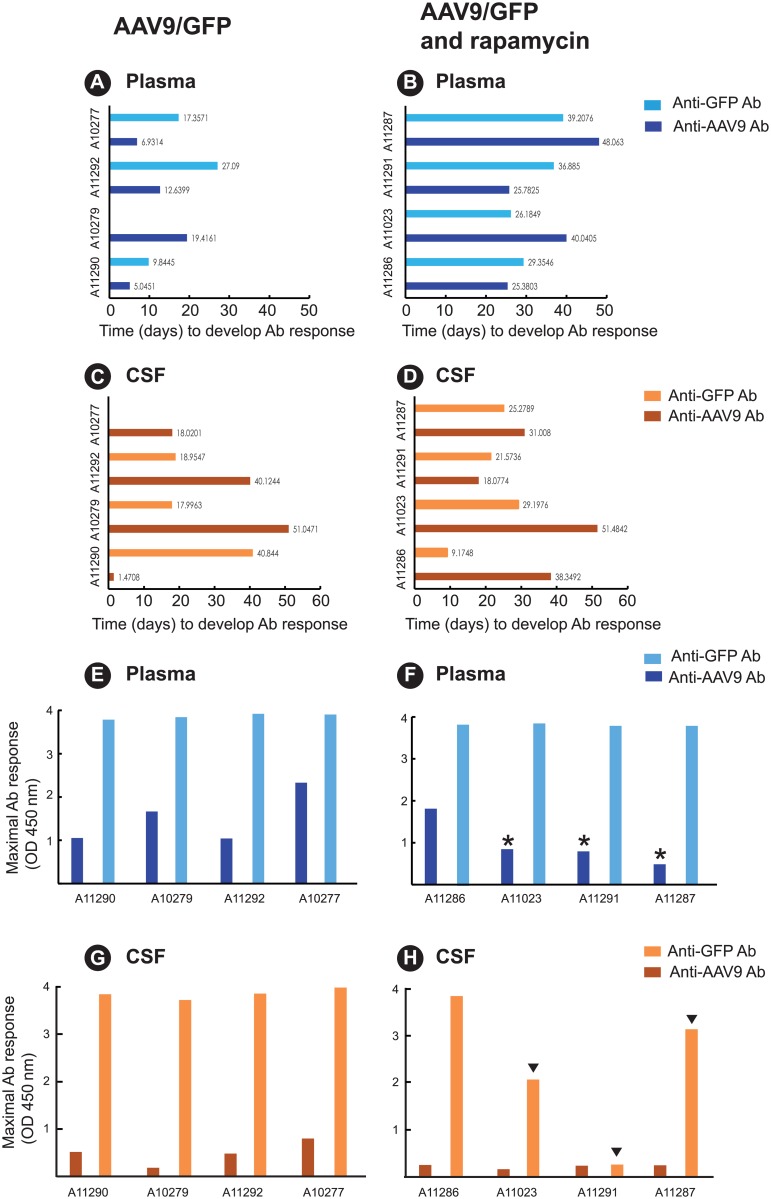
Treatment with rapamycin delays the time to peak GFP and AAV9 antibody (Ab) responses and the magnitude of the peak antibody response. The estimated Delay parameter (time before change is observed) is plotted in panels A and B (Ab responses in plasma) and panels C and D (Ab responses in CSF) for the two treatment groups: AAV9/GFP (controls) and AAV9/GFP + rapamycin. Rapamycin delayed the time to detectable plasma AAV9 capsid Ab (P<0.005) and to detectable plasma GFP Ab (P<0.025). The observed Maximal Ab response (observed maximum optical density (O.D.) value of all observations) for each group is plotted in panels E and F (Ab response in plasma) and panels G and H (Ab response in CSF). Asterisks indicate animals exhibiting reduced AAV9 capsid Ab response after rapamycin treatment (panel F) relative to levels observed in the four controls. Arrowheads indicate animals exhibiting reduced GFP Ab responses after rapamycin treatment (panel H).

To further examine the suppressive effects of rapamycin on the antibody response, we compared delay in development of detectable antibody (estimated) (Fig 2A–2D) and the maximum antibody response (Fig 2E–2H). The time to first detection of AAV9 capsid antibody or GFP antibody in plasma in the control group was 5-to-19 days and 10-to-27 days, respectively (Fig 2A). In contrast, the rapamycin group (Fig 2B) exhibited a significant delay of 25-to-48 days in the time to detectable plasma AAV9 capsid antibody (P<0.005) and a delay of 26-to-39 days in the time to detectable plasma GFP antibody (P<0.025). In the CSF, there was no difference in time to detectable AAV9 capsid antibody or GFP antibody between the controls (Fig 2C) and the treatment group (Fig 2D).

Treatment with rapamycin did not affect the maximal GFP antibody response, defined as the observed maximum value of all measurements, in plasma when compared to AAV9/GFP only controls (Fig 2E and 2F). However, treatment with rapamycin reduced the maximal capsid antibody response in the plasma in three of the four animals (Fig 2F) when compared to responses observed in the four AAV9/GFP only control animals (Fig 2E). A comparison of the mean maximum anti-capsid antibody response in the plasma between these two groups did not reach statistical significance (S2 Table, P = 0.200).

Treatment with rapamycin reduced GFP antibody responses in the CSF in three of four animals, although the mean antibody responses fell short of statistical significance (P>0.086) when compared to the AAV9/GFP only controls (Fig 2G and 2H). CSF AAV9 capsid antibody remained low in both groups. Taken together, these results, summarized in S2 Table, indicate treatment with rapamycin delayed and/or suppressed the development of GFP and AAV9 capsid-specific antibody responses in the plasma and CSF.

### Cytokine profiles in the CNS are altered with rapamycin treatment

The kinetics of cytokine expression was measured in plasma and CNS by Bioplex analysis of 26 cytokines and chemokines at 0, 14 and 56 days post-treatment. The multiplex data was analyzed using a linear model least squares regression analysis. A positive slope indicates an increase in cytokine expression (S2 Fig). Of 26 biomarkers, 12 showed changes in expression while 14 showed no change in expression in the CSF of either group (Table 1). In the rapamycin group, all 12 cytokine/chemokine biomarkers showed increased expression in the CSF (Table 1 and S2 Fig). In the control group, only four cytokines (sCD40L, IL1ra, IL13, and IL12) of the 12 increased in the CSF. Notably, eight distinct T cell suppressive or immunostimulatory biomarkers exhibited increased expression in the CSF and were unique to the rapamycin group (S2 Fig). These included IL10, IL2, IL5, IL15, VEGF, IL6, G-CSF, and IL8 (Table 1). There was no change in the expression of the 26 biomarkers in the plasma of either the rapamycin or control groups (S2 Fig). In summary, these results show that AAV9/GFP inoculation had limited effects on certain inflammatory or regulatory biomarkers but only in the CSF. When compared to the AAV9/GFP controls, rapamycin treatment increased a broader range of both inflammatory and regulatory biomarkers in the CSF but not in the plasma.

**Table 1:** Rapamycin alters the cytokine profile in the cerebrospinal fluid after gene delivery.

Cytokine	Function	CSF	
		AAV9/GFP	AAV9/GFP and rapamycin
IL10	Regulatory; anti-inflammatory; secreted by T regs	no change	increased
IL2	Growth factor T cells (T regs compete strongly)	no change	increased
sCD40L	Promotes T regs, MDSC	increased	increased
IL1ra	Inhibits proinflammatory effects of IL1	increased	increased
IL5	TH2; growth and differentiation factor for B cells and eosinophils	no change	increased ^q^
IL13	TH2; growth and maturation of B cells; inhibits proinflammatory cytokines	increased	increased
IL12p40	Growth factor for activated T and NK cells	increased	increased
IL15	Growth factor for T and NK cells; proliferation of memory CD8 T cells	no change	increased
VEGF	Growth factor for endothelial cells and in angiogenesis, vasculogenesis	no change	increased
IL6	Proinflammatory; lymphocyte and monocyte differentiation; TH17	no change	increased
G-CSF	Induces granulocytes	no change	increased
IL8 (CXCL8)	Neutrophil activation	no change	increased

Comparisons between groups were determined based on using a linear model least squares regression analysis. Polynomial regressions were applied to the cytokine responses over time and only statistically significant results are highlighted as shaded blocks. All increases are linear except where noted by ‘q’.

^q^; cytokine expression is a quadratic function.

### GFP-specific T cell responses are reduced after rapamycin treatment

**Fig 3:**
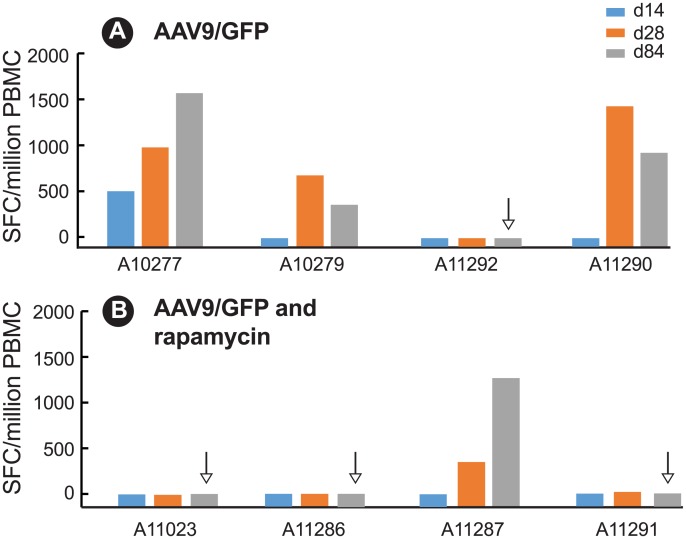
GFP-specific T cell responses as spot forming cells (SFC)/10^6^ PBMC after rapamycin treatment. T cell responses in PBMC were measured by ELISPOT at 14, 28, and 84 days after AAV9/GFP delivery in (A) AAV9/GFP only (controls); (B) AAV9/GFP + rapamycin. Arrow indicates undetectable (< 10 SFC/10^6^ PBMC) GFP-specific responses at necropsy (84 days).

Consistent with antibody responses, GFP was more immunogenic than the AAV9 capsid and induced stronger T cell responses in the periphery as measured by ELISPOT analysis of IFN-γ T cell responses in peripheral blood mononuclear cells (PBMC). Moderate-to-strong GFP-specific T cell responses, ranging from 115 to 1672.5 SFC/million PBMC, were detected in 4 of the 8 macaques (Fig 3) while AAV9-specific responses were negligible in all animals with only one animal (A11290) in the control group exhibiting a low AAV9-specific T cell response (72.5 SFC/million). In the AAV/GFP only controls, GFP-specific T cell responses peaked between 28 and 84 days in 3 of the 4 animals after transgene delivery (Fig 3A) but were low (< 500 spot forming cells) to undetectable in 3 of the 4 animals in the rapamycin (Fig 3B) group. At necropsy, PBMC from 3 of 4 animals in the rapamycin group (Fig 3B) were negative for GFP-specific T cell responses, compared to only one macaque in the AAV9/GFP only control group (Fig 3A).

### Rapamycin treatment alters CD4 and CD8+ T cell effector functions

**Fig 4:**
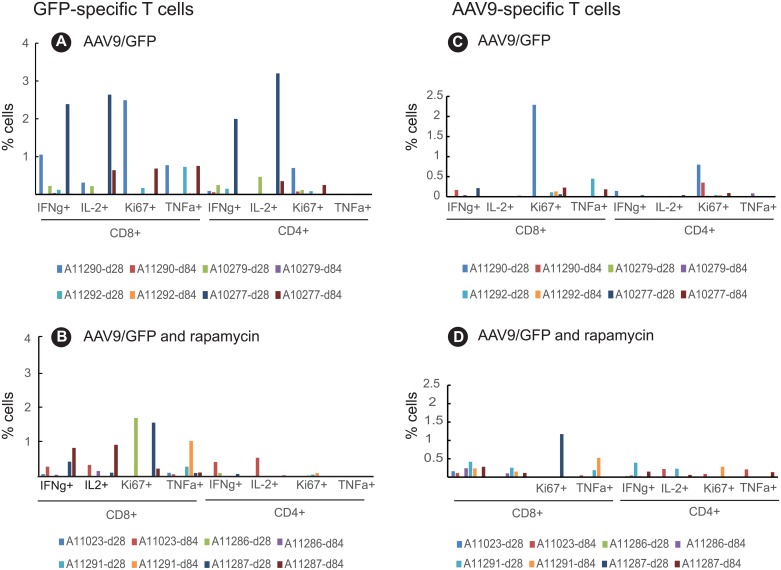
Magnitude and breadth of GFP-specific T cell responses reduced after treatment with rapamycin. Multiparameter flow cytometry to assess T cell responses was performed using peptide (GFP or AAV9 peptide pools)-stimulated PBMC. PBMC were stained with antibodies to detect the following markers: CD4, CD8, IFN-γ, IL2, Ki67, and TNFα. Panels A and B depict GFP- specific T cell responses in two treatment groups: AAV9/GFP (controls) and AAV9/GFP + rapamycin. Panels C and D depict AAV9-specific T cell responses.

**Fig 5:**
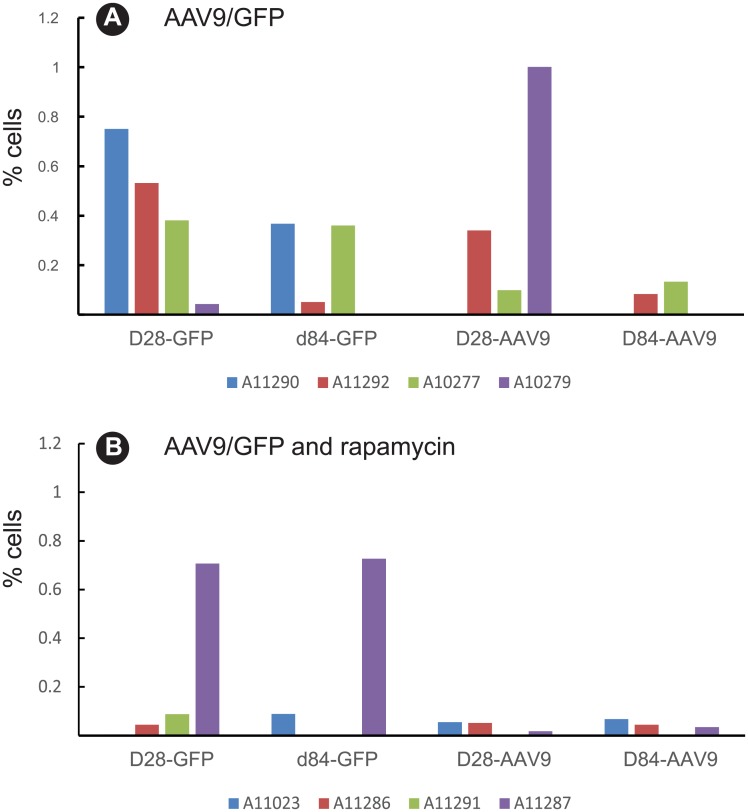
Cytotoxic GFP-specific T cells decreased after rapamycin treatment. Multiparameter flow cytometry was used to identify CD8+ CD107+ T cells in PBMC after stimulation with GFP peptide pools. CD8+CD107+ expression in PBMC was monitored 28 and 84 days after transgene delivery in (A) AAV9/GFP (controls); (B) AAV9/GFP and rapamycin.

The magnitude and function of T cell responses induced were assessed by measuring CD4+ and CD8+ T cell expression of the cytokines IFN-γ, IL-2, TNF-α as well as Ki67 (marker of proliferation) and CD107a (a degranulation marker of cytolytic T cells) by intracellular cytokine staining (ICS) and flow cytometry following *in vitro* stimulation with overlapping peptides encompassing GFP or the AAV9 capsid. GFP-specific CD4 and CD8 T cell responses in the peripheral blood of the AAV9/GFP control group peaked 28 days after transgene delivery whereas AAV9 responses were not significantly different from levels measured at baseline (Panels A and C in S3 Fig). GFP-specific T cell responses that developed in the AAV9/GFP control group were multifunctional as evidenced by the production of multiple cytokines and as indicated by the frequency of T cells expressing one or more cytokines (Fig 4A) or CD107a (Fig 5A) following GFP peptide stimulation. Depending on the cytokine measured, CD4+ and CD8+ GFP-specific T cell responses at 28 days post-inoculation in the AAV9/GFP control group (Fig 4A) peaked and ranged from 0.1–3.2% and tended to be higher when compared to the rapamycin group that exhibited responses ranging from undetectable (0)– 1.9% (Fig 4B). Overall, GFP-CD4+ T cells but not GFP-CD8+ T cells expressing IFN-γ, IL-2, Ki67 and/or TNF-α were significantly suppressed in the rapamycin group when compared to the AAV9/GFP only controls (P = 0.0286) (S4 Fig). AAV9-specific T cell responses in the control (Fig 4C) and rapamycin (Fig 4D) groups were weak and exhibited limited functions. Furthermore, T cell activation measured by expression of Ki67 on CD4 and CD8+ T cells was low in both control and rapamycin treated groups.

Cytolytic GFP and AAV9-specific CD8+ T cells were measured by flow cytometry analysis of CD107a expression, a marker of degranulation following *in vitro* stimulation with overlapping GFP or AAV9 peptides (Fig 5). Three animals in the control group (AAV9/GFP) exhibited significant cytolytic GFP-specific (A11290, A11292, and A10277) or AAV9-specific T cell responses (A11292, A10277, and A10279) (Fig 5A). In contrast, none of the animals in the rapamycin group developed detectable cytolytic AAV9-specific T cells and only one animal in this group (A11287) had detectable GFP-specific T cells (Fig 5B). These results indicate rapamycin treatment suppressed development of effector T cell responses against the GFP transgene.

### Sustained GFP expression in the lumbar spinal cord after rapamycin treatment after 12 weeks

**Fig 6:**
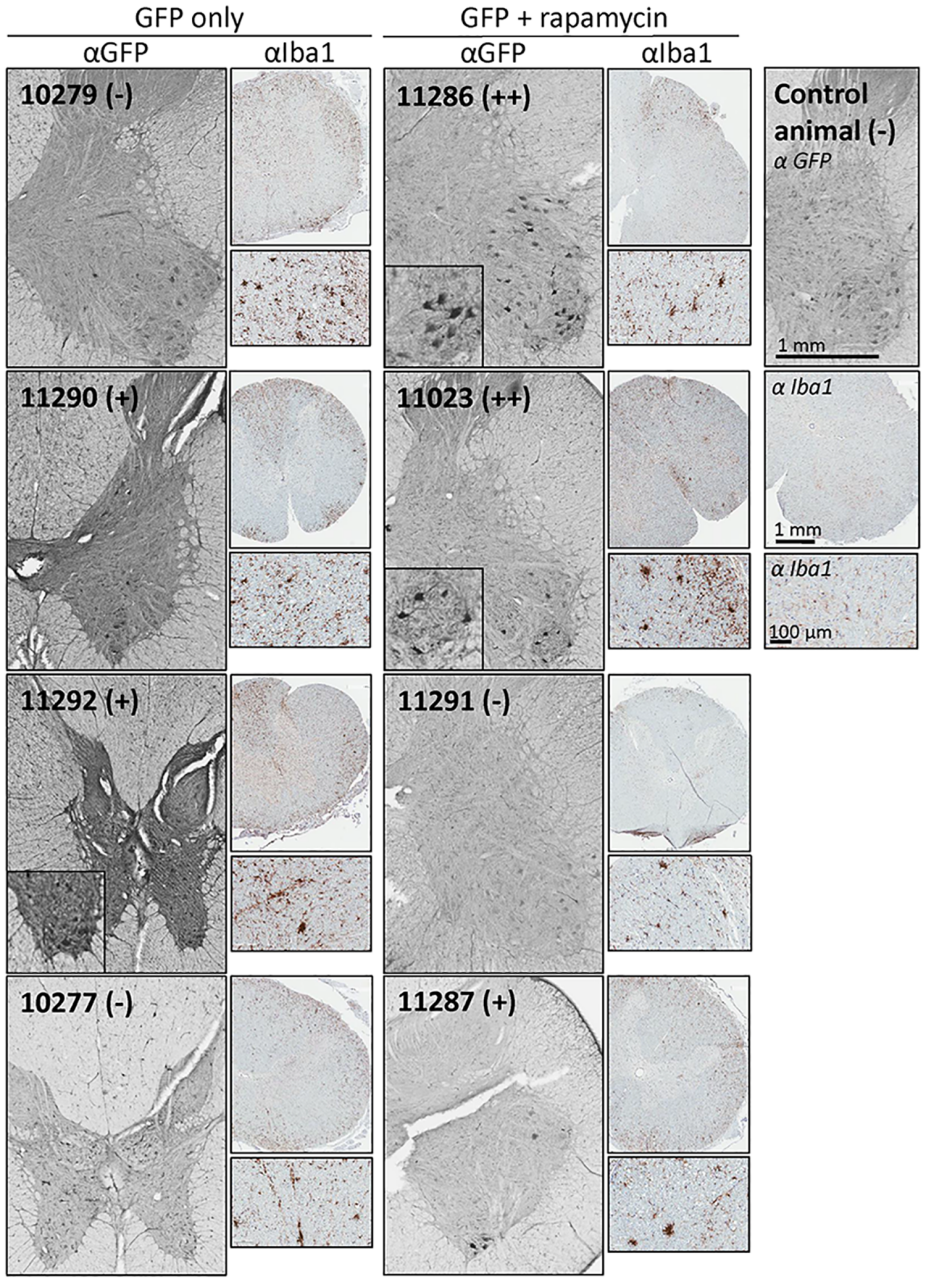
IHC was carried out to visualize GFP expression in the lumbar spinal cord. Shown are representative 40 micron lumbar spinal cord sections of all study macaques, stained for GFP. Magnified insets show examples of GFP-positive motor neurons. Macaque ID numbers are provided in each panel, along with a qualitative scoring of ventral horn expression. The (-) indicates no GFP-positive staining above that seen in uninjected NHPs; (+) indicates some positive cells observed, above that seen in uninjected macaques; (++) indicates relatively strong expression in a large number of neurons.

To assess the potential impact of the anti-GFP immune responses on transgene expression, protein expression at 12 weeks was assessed by immunohistochemistry (IHC) for GFP within the neurons in the ventral horn of the spinal cord. Table 2 shows the summaries of all sections sampled, while Fig 6 shows representative fields. GFP-positive neurons were most robust in the animals treated with rapamycin. Two macaques in this group showed strong GFP expression (Fig 6 insets—similar fields were not observed in the controls) while one NHP showed moderate expression. In contrast, two macaques in the control group showed no expression, while two showed moderate GFP expression; none of these animals showed the number of strongly-labeled cells seen with two within the treated group. These data indicate treatment with rapamycin is able to enhance or prolong expression of the GFP transgene. Additional IHC showed enhanced Iba1 expression indicative of microglial activation at the time of necropsy in all injected animals. There was no clear correlation between Iba1 staining and GFP expression, and the increased Iba1 reactivity was mostly localized to the white matter (S5 Fig). However, all samples are from the endpoint of the experiment, which was designed to look for persistent GFP expression. The most critical times to directly assess inflammation may be earlier, at times closer to when the rapamycin was discontinued.

**Table 2:** GFP expression in lumbar spinal cords of NHPs monitored by IHC.

Treatment Group	Animal ID	GFP Expression (IHC)
AAV9/GFP (controls)	10279	-
	11290	+
	11292	+
	10277	-
AAV9/GFP + rapamycin	**11286**	**++**
	**11023**	**++**
	11287	+
	11291	-

(-) indicates no GFP-positive staining above that seen in uninjected NHPs

(+) indicates some positive cells observed, above that seen in uninjected NHPs

(++) indicates relatively strong expression.

## Discussion

Replacement of mutant or null protein that is critical to normal physiological function is an obvious application of gene therapy. This process has been limited by the effects of the immune system, which can act to aggressively clear "novel" proteins at varying times after delivery. Immune tolerance and/or immune suppression are therefore essential to promote long-term therapeutic transgene expression [8]. This is true even for direct delivery of vectors within the CNS. Tardieu et al [9] investigated a human gene replacement using intracerebral AAV2/5 plus oral tacrolimus and mycophenolate mofetil for treatment of mucopolysaccharidosis Type IIIB syndrome in young children; all showed improvement and evidence of immunological tolerance. Doerfler et al [10] also discussed this issue in detail and published data using a co-packaged AAV9 system, combining a liver-specific promoter with a tissue-restricted desmin promoter to express the curative transgene and induce immune tolerance in a mouse model of Pompe disease where the correction is systemic [11].

The present study was undertaken to investigate potential modifications that could be incorporated into the clinical trial protocol for giant axonal neuropathy (GAN) (ClinicalTrials.gov identifier NCT02362438), a rare neurodegenerative autosomal recessive disease[12], with implications for other gene therapy trials. The original inclusion criteria of the trial required enrolled patients to have at least one gigaxonin gene allele with a missense mutation so that they would have expected pre-existing tolerance to the gigaxonin protein. However, there are patients for GAN and other gene replacement trials that could not be inherently self-tolerant to the expressed therapeutic protein (“null” patients such as homozygous nonsense mutations or deletions). The GAN clinical protocol utilizes the same AAV9 vector that was used to express GFP in this study. The clinical AAV9/GAN vector, scAAV9/JeT-GAN [13] is administered by intrathecal delivery to the brain and spinal cord of patients with GAN, and is the first-in-human trial of intrathecal delivery of this gene transfer vector. The assumptions are (a) that patients with missense mutations in the GAN gene may be immunologically tolerant to gigaxonin and (b) that immune responses to the AAV9 would be minimal or manageable, so that they have little or no impact on transgene expression. However, null patients are unlikely be immunologically tolerant to gigaxonin or the AAV9 vector, and could mount an adaptive immune response that clears transduced cells. The present study was undertaken to address the hypothesis that by decreasing immune reactivity before and within the first few weeks after intrathecal delivery of a transgene by AAV9, improved expression of the transgene would occur in the CNS. GFP was chosen as the transgene because it encodes a strong immunogen and models a ‘worse-case’ scenario for a transgene-specific immune response. Here, we show that in a non-human primate model, a modulatory dose of the immunosuppressant drug rapamycin reduced the overall immunogenicity of AAV9/GFP by decreasing T cell responsiveness to GFP, and by lowering GFP- and AAV9-specific antibody responses. Furthermore, reduced GFP-specific T cell responses in the rapamycin treated group corresponded to sustained gene expression in the CNS for twelve weeks after transgene delivery.

We also observed no evidence of toxicity due to either the therapy itself, or in the experimental group that also received rapamycin. This agrees with a previous study by one of our group, using a strong CBh promoter to drive GFP [14] as well as a study by Hinderer et al [15] where AAV9-GFP was injected directly into the cisterna magna. There is one previous report that described a strong cytotoxic lymphocyte (CTL) response against GFP in cynomolgus macaques injected with an AAV9/GFP vector into the cisterna magna [16]. Animals in this study developed cerebellar damage and ataxia requiring euthanasia at approximately 3 weeks post-injection [16]. These effects were not seen in animals receiving an AAV9 vector expressing a representative self-antigen, aromatic L-amino acid decarboxylase (AAV9/AADC), indicating the anti-GFP response in that study was the causal factor rather than an anti-AAV9 capsid response. In contrast, we observed no deleterious symptoms in animals inoculated with AAV9/GFP into the CSF for the 12-week duration of the study. This difference could be attributed to a number of variables in our study including the use of the JeT promoter that results in minimal expression of GFP, administration of a lower dose of AAV9/GFP when compared to the Samaranch *et al*. study [16], differences in route of delivery, or differences in the AAV9 vector production/ purification methods. Even though none of the macaques in our study exhibited deleterious symptoms, the induction of significant GFP-specific immune responses in animals that received AAV9/GFP alone demonstrates that immune responses can develop following AAV-mediated transfer of an immunogenic foreign gene into the CNS.

Following CNS delivery of AAV9/GFP, cytokine responses in the periphery and the CNS were compartmentalized. Twelve of the 26 cytokines or chemokines measured 2 and 8 weeks after inoculation were detected in the CSF whereas none was detected in the plasma. This compartmentalization is likely due to the barrier function of the blood-brain barrier (BBB) and the blood-CSF barrier. Although AAV9/GFP was delivered into the CSF, AAV9 capsid and GFP antibodies were detected in both plasma and CSF of both groups, a result that may be due to some leakage of AAV9/GFP into the peripheral circulation during the intrathecal delivery. Notably, there was also compartmentalization in the kinetics and magnitude of the antibody response to GFP and the AAV9 capsid in plasma and CSF. In the control group, GFP and anti-AAV9 responses appeared earlier and were stronger in plasma, an outcome that may be due to a more robust immune environment in the blood. Although GFP is expressed intracellularly in the CNS, strong GFP-specific antibody responses were induced. This may be due to GFP secretion, apoptosis of GFP expressing cells or release of GFP via exosomal pathways, which have been recently identified in the CNS[17].

Rapamycin targets mTOR, a serine/threonine protein kinase that is evolutionarily conserved and constitutively expressed[18]. The mammalian target of rapamycin (mTOR) is a central regulator of immune responses because of its role in sensing and integrating cues from the immune microenvironment and impacts multiple immune functions, including B cell differentiation, activation, and function. Consistent with this function, macaques treated with rapamycin exhibited delayed time to development of anti-GFP and anti-capsid responses in plasma, as well as reduced magnitude in the antibody response in plasma (anti-capsid) and in CSF (anti-GFP). These results show that rapamycin effectively dampened but did not prevent development of antibody responses to both the AAV9 vector and the GFP transgene likely via its effects on B cell development and function in both the periphery and CNS.

Attempts to monitor AAV9- or GFP-specific T cell responses in the CNS were unsuccessful due to insufficient number of cells in the CSF after transgene delivery. Instead, cytokine profiles in CSF and plasma were analyzed as markers of inflammatory or immune regulatory responses. Again, we observed a compartmentalization of the cytokine response with more cytokine/chemokine increases detected in the CSF than in the plasma or distinct cytokine profiles in plasma versus CSF. With rapamycin, growth factors and cytokines that function in anti-inflammatory or pro-inflammatory responses increased in the CSF, including the main anti-inflammatory cytokine, IL10. IL10 is a pleiotropic cytokine, promoting neuronal and glial cell survival and dampening inflammatory responses via multiple signaling pathways[19]. IL10 ultimately ends the inflammatory response by reducing activation and effector functions of T cells, monocytes, and macrophages[19, 20]. Additional anti-inflammatory cytokines detected in the CSF of NHP treated with rapamycin include IL1ra [21], IL13 [22], sCD40L [23], and IL12-p40 [24]. The IL12-p40 subunit may dimerize with alpha chains, p35 or p19, to form IL12 or IL23, respectively; and in this form, can promote inflammatory responses [24]. However, IL12-p40 can also be secreted independently as a monomer or as a disulphide-linked homodimer and instead, promote anti-inflammatory responses [24]. The anti-inflammatory cytokines, IL1ra, IL13, sCD40L, and IL12-p40, were also increased in the control group but not the main anti-inflammatory regulator, IL10, which suggests rapamycin may promote induction of IL10 expression. Proinflammatory cytokines were also detected in the CSF in the rapamycin group. These include IL6 and IL8 which activate microglia [22]. The paradoxical observation that rapamycin induced the immunoregulatory cytokine, IL10, and the immunostimulatory cytokines, IL6 and IL8, is a known consequence of inhibiting mTOR signaling[25]. The underlying mechanisms of such contradictory effects on different immune cell types are not fully understood and may depend partly on the levels of mTOR signaling. Growth factors detected in the rapamycin group include IL2, IL5, IL15, and G-CSF and suggest the presence of activated T cells (IL2) [26], B cells (IL5) [27], memory CD8 T cells (IL15) [28] and neutrophils (G-CSF) [29] in the CSF. However, frequencies of these cells must be low since there were insufficient numbers of cells in the CSF for flow cytometry analysis, and inflammatory infiltrates were not observed in brain tissues at necropsy. Together, these data show that rapamycin treatment had a pleiotropic effect in increasing a range of biomarkers in the CSF including both regulatory responses as well as immunostimulatory responses. The precise mechanisms and effects of biomarker modulation, especially those with competing function, in the CSF in this group are not known. However, enhanced expression of local regulatory biomarkers may reflect mechanisms by which rapamycin dampens immune responses.

In summary, we show that short term treatment with rapamycin one week before and for two weeks after administering AAV9/GFP decreased immune reactivity of both the AAV9 vector and an immunogenic model antigen, GFP, and resulted in sustained expression of the transgene in the CNS. These findings support the use of rapamycin to dampen immune reactogenicity of AAV9-based gene therapies in human clinical trials. The duration of the immunosuppressive effects with short-term rapamycin treatment will require additional long-term studies (longer than 12 weeks) to further optimize the regimen and dosing of rapamycin needed to achieve maximum benefit with minimal risk. Follow-up risk-benefit studies should include AAV9 dose vs. distribution (higher and lower), longer-term dosing with rapamycin or analogs, and histopathological assessment of more acute localized inflammatory responses.

These studies certainly have relevance beyond gene replacement therapies. Genome editing technologies are being increasingly considered as realistic human treatments, but editases such as Cas9 (a bacterial protein) would be viewed as a foreign antigen to the human immune system. It might be favorable to express these editases at low levels similar to the design of the JeT-GAN vector, and our data support the notion that rapamycin could be used to manage deleterious immune response to editases or other foreign proteins.

There is increasing evidence that long-term persistence of transgenes will require immune tolerance when the transgene expresses a foreign antigen, possibly even in cases where the novel aspects of the protein structure appear to be subtle (e.g., missense mutations) and the gene has been delivered within the BBB. Infections or other environmental exposures later in life can potentially trigger a delayed response that is initially latent, and could result in the clearance of neurons and other cells that are expressing the therapeutic protein. Our findings support further investigation of rapamycin to modulate and reduce these events, and indicate that for both short and long-term efficacy, modulation of the immune response to gene therapy is likely to have significant clinical benefits.

## Materials and methods

### Animals

All animal experiments used in this study were approved by the University of Washington Institutional Animal Care and Use Committee (UW protocol #4266–04), and were in compliance with the U.S. Department of Health and Human Services Guide for the Care and Use of Laboratory Animals and Animal Welfare Act. This study used male cynomolgus macaques (*macaca fascicularis*) between the ages of 4 and 6 (average 5). Animals were originally obtained from approved breeding facilities and vendors (California National Primate Research Center or SNBL, Everett, WA) and were pre-screened for cross-reactive antibody to AAV9 prior to enrollment in the study by ELISA. AAV9/GFP doses were diluted in 1ml saline and injected intrathecally. Rapamycin (Sirolimus, Pfizer) was obtained in tablet form, crushed, dissolved in DMSO and doses were administered orally one time daily by mixing with palatable food (yogurt or fruit). A dose of 0.5mg/kg was administered on day one and then maintained at 0.2mg/kg for 20 days thereafter for a total of 21 days of rapamycin dosing. Animals were monitored to confirm entire dose was consumed. Doses were administered at the same time each day. At each sampling time-point, 10–20 ml of blood was collected by venipuncture and 0.5–1 ml cerebral spinal fluid (CSF) was collected by lumbar puncture. Animals were singly housed in an AAALAC-accredited facility. Cages, racks, and accessories were sanitized in mechanical cage washers at least once every two weeks and were cleaned with water daily. Temperature in animal quarters was maintained at 72–82°F. Animals were fed a commercial monkey chow, supplemented daily with fruits and vegetables and drinking water was available at all times provided by automatic watering devices. Throughout the study, animals were checked twice daily by the veterinary technicians to evaluate their physical and clinical condition. Environmental enrichment activities included grooming contact, perches, toys, foraging experiences and access to additional environment enrichment devices such as paint rollers, grooming devices, foraging devices, activity panels and mirrors. All procedures were performed under ketamine sedation (10 mg/kg) or Telazol (2.5–5 mg/kg) to minimize pain. Euthanasia prior to necropsy was performed by administration Euthanol^®^ (Virbac Corp., Houston, TX) while the animal was under deep anesthesia in accordance with guidelines established by the 2007 American Veterinary Medical Association Guidelines on Euthanasia which is consistent with the guidelines described in the Weatherall Report on The Use of Nonhuman Primates in Research. None of the animals became severely ill during the course of the study and none required euthanasia prior to their experimental endpoint.

### Vector production

The AAV9 vectors used in these studies packaged a self-complementary (sc) genome. For the scAAV9/JeT-GFP vector, GFP expression mediated by the synthetic JeT promoter and synthetic polyA[30]. AAV vectors were produced using methods developed by the University of North Carolina Vector Core facility, as described [31]. In brief, the production plasmids were triple-transfected into suspension HEK293 cells. AAV vectors were purified from the cells by iodixanol gradient centrifugation, followed by ion-exchange chromatography. The purified AAV was dialyzed in PBS supplemented with 5% D-Sorbitol and an additional 212 mM NaCl (350 mM NaCl total). The titer was determined by quantitative PCR and confirmed by polyacrylamide gel electrophoresis (PAGE) and silver stain. Quality control measures were in place that the qPCR titer and PAGE/silver stain titer match within 2-fold, that no contaminating proteins are visible by PAGE, and that the viral capsid proteins migrate at the expected size with a 10:1:1 VP3:VP2:VP1 ratio.

### Study design

To determine if gene therapy using an AAV9 vector could be tolerized, we administered an AAV9 vector expressing a model gene, GFP (AAV9/GFP), into cynomolgus macaques (N = 4 per group) via the intrathecal route either with or without rapamycin at an immunomodulatory dose. AAV9/GFP + rapamycin was compared to a control group receiving AAV9/GFP alone (S1 Fig). Macaques received a total of 8.5 x 10^12^ vg of AAV9/GFP. Rapamycin (R&D Systems) was administered daily to the two test groups at a dose of 2mg/kg per day for one week prior to AAV9 inoculations and continued for 2 weeks (14 days) at a dose of 1mg/kg following the AAV9 inoculations. Doses of AAV9 and rapamycin were based on prior effective doses for these treatments in nonhuman primates or in humans. Blood, CSF, and axillary lymph nodes were collected at 2–4 week intervals to analyze immune responses (antibody and T cell responses) and blood chemistries. In addition, lymph node, brain, spinal cord and dorsal ganglia tissues were collected at necropsy (week 12 post-AAV9 inoculation) to determine if the treatments induced neural injury or inflammation.

### Antibody detection by ELISA

GFP and AAV9 capsid-specific IgG antibody levels in macaque serum and CSF were assessed by ELISA. Maxisorp plates (Thermo Scientific-Nunc) were coated with 100 ng/well of either recombinant GFP or AAV9 capsid in PBS overnight at 4°C. Plates were blocked with 5% nonfat milk powder in PBS for 1 h at room temperature, and then washed three times with wash buffer (PBS-T; phosphate-buffered saline containing 0.05% Tween 20). Three-fold serial dilutions of samples were added to the wells, and plates were incubated for 1 hr at room temperature. Following three washes with PBS-T, plates were incubated with horseradish-peroxidase conjugated goat anti-macaque IgG (1/5,000 dilution) secondary antibodies (Nordic Immunological Laboratories) for 1 hr at room temperature. After five washes with PBS-T, TMB substrate (KPL) was added to the wells for 30 min at room temperature. Color development was stopped by the addition of TMB Stop solution (KPL), and the plates were read at 450 nm.

### Multiplex cytokine assay

The concentrations of 23 cytokines in plasma and CSF were measured on days 0, 14 and 56 days post-inoculation of the AAV9 vectors using a Bio-Plex multiplex bead array kit (Bio-Rad, Hercules, CA). The Bio-Plex assay was performed in accordance with the manufacturer’s instructions.

### IFN-γ ELISPOT assay

Isolated peripheral blood mononuclear cells (PBMC) were stimulated with individual peptide pools (1μg/mL each peptide) spanning the full amino acid sequences of the AAV9 capsid or GFP. The peptides were 15-mers overlapping by 11 amino acids (BEI Resources). Concanavalin A (Sigma) was used as a positive stimulation control (5μg/mL). DMSO served as a negative solvent control. Antigen-specific T cells secreting IFN-γ were detecting using paired anti-macaque IFN-γ monoclonal antibodies (U-cytech-BV) as previously described. Spot forming cells (SFC) were enumerated using an Immunospot Analyzer with CTL Immunospot Profession Software (Cellular Technology Ltd.). Results are expressed as the mean number of SFC in replicate wells containing antigenic peptide, subtracting the number of spots from DMSO control wells from the same animal.

### Intracellular cytokine staining and flow cytometry

Multiparameter flow cytometry to determine T-cell immune responses was performed using peptide (GFP or AAV9 peptide pools)- stimulated PBMC as previously described (50). One million PBMC were stained for each condition (DMSO, PMA/Ionomycin, and peptide(s)) for 10–14 hours. PBMC were stained with the following antibodies Live/Dead Yellow (Invitrogen^®^), CD3-APC (BD Biosciences, clone SP34-2), CD4-PerCP Cy5.5 (BD BioSciences, clone L200), CD8 APC-Cy7 (BD, clone RPA-T8), IFNγ-BV650 (BioLegend, clone 4S.B3), IL-2-PE (BioLegend, clone MQ1-17H12), TNFα-PECy7 (BD, clone Mab11), CD107a-FITC (BD, clone H4A3). Cells were fixed in 1% paraformaldehyde and acquired using an LSR II flow cytometer (BD Biosciences) and the data were analyzed using FlowJo software (Tree Star, Inc., Ashland, OR). Samples were considered positive if peptide-specific responses were at least twice that of the negative control plus at least 0.01% after background subtraction.

### Evaluation of GFP expression in the CNS

To directly assess GFP expression by histology, IHC against GFP was conducted. After one week of fixation in PBS with 4% paraformaldehyde, the lumbar spinal cords were sectioned at 40 microns using a Leica vibrating microtome at room temperature. Samples were incubated in 3% H_2_O_2_ for 30 minutes and then incubated for 1 hour at room temperature in blocking solution (10% goat serum, 0.1% triton X-100, 1X PBS), then incubated for 24 hours at 4°C in primary antibody solution (5% goat serum, 0.1% triton X-100, 1X PBS, rabbit anti-GFP [Millipore # AB3080, 1:1000]). After washing 5 times 5 minutes each in 1X PBS, samples were incubated in ImmPRESS anti-Rabbit detection reagent (Vector Lab, #MP-7401) for 2 hours at room temperature. Then after washing 3 times 5 minutes each in 1xPBS, 3,3’-diaminobenzidine tetrachloride substrate (DAB, ACROS Organices, #7411-49-6) and nickel-cobalt solution was used to develop the reaction product. Sections were imaged using an Aperio ScanScope XT system (Aperio Technologies) and viewed using ImageScope software (v. 12.3.1; Aperio Technologies).

It should be noted that the JeT promoter utilized in these studies only confers minimal expression, which created technical difficulties assessing transgene expression by IHC. Consistent with previous reports[15], even after optimization of IHC conditions a low level of non-specific staining was seen in lumbar spinal cord sections from naïve uninjected animals (S5 Fig, “control animal” panel). However, GFP mRNA transcripts were not detected in these controls by RT-qPCR. Qualitative visual scoring of expression was carried out by reviewing at least 8 sections from each animal. Sections from 3 naïve NHPs were stained in parallel as negative controls and reviewed to determine background levels of non-specific staining.

### Evaluation of Iba1 expression in the CNS

5 μm monkey lumbar cord paraffin sections were used for Iba1 IHC staining. Slides were deparaffinated by xylene and rehydrated with gradient ethanol and water, and washed with 1xPBS twice for 3 minutes each. Antigen retrieval was performed with antigen unmasking solution (AUS, Vector H-3300) using a pressure cooker for 5 minutes, and washed with tap water and 1xPBS. Then slides were incubated with 3% H_2_O_2_ for 30 minutes at room temperature, washed with tap water and 1xPBS, and then blocked with 5% goat normal serum (GNS) for 1 hour at room temperature. Tissues were blocked with Avidin solution (Vector Labs SP2001) for 15 minutes and rinsed with 1xPBS briefly, then blocked with Biotin solution for 15 minutes and rinsed off. Next, slides were incubated with Anti-Iba1, Rabbit (Wako, 019–19741), diluted 1:1000 in GNS, at 4 °C overnight. On the second day, after washing with tap water and 1xPBS, slides were incubated with biotinylated anti-rabbit secondary antibody (Vector Labs BA-1000) 1:200 for 1 hour at room temperature. Slides were washed and incubated with ABC (Vector Labs PK-6100) for 30 minutes at room temperature and washed with water and 1xPBS. 3,3’-diaminobenzidine tetrachloride substrate (DAB, ACROS Organices, #7411-49-6) was used to develop the reaction product. Finally, tissues were counter stained with Meyer’s hematoxylin. Sections were imaged as described above.

### Statistical analysis

Logistic regression analysis was used to model the antibody data obtained by ELISA. Three estimated parameters (background, slope, and delay) were obtained by non-linear least squares. Background = Estimated value at Time = Delay. Slope = Estimated slope (this characterizes the direction of the response). Delay = Estimated time before a change is observed. Non-linear least squares also produces a Residual Sum of Squares (RSS) as a measure of how well the estimated model fits the responses. A small RSS indicates a tight fit of the model to the data. In this analysis, RSS values ranged from 0.05 to 0.1, which suggest that the estimated model is a good fit of the data.

The multiplex cytokine data was analyzed using a linear model least squares regression analysis. Polynomial regressions were applied to the cytokine responses over time and only statistically significant terms retained. In the majority of cases (83%), no time dynamics were detected. For those cytokines whose expression changed, the increase was either linear or quadratic (transient increase followed by decrease).

Comparisons of mean immune responses between two groups was analyzed by Mann-Whitney U test and between groups by one-way ANOVA.

## Supporting information

S1 Fig Study design. Cynomolgus macaques (N = 4 per group) were randomized into two treatment groups, AAV9/GFP only (control group), and AAV9/GFP with rapamycin (rapamycin group). For both groups, AAV9 vectors were administered intrathecally. Rapamycin was administered orally on a daily basis for one week prior to AAV9 inoculations and continued for 2 weeks following the AAV9 inoculations. Blood, CSF, and lymph nodes were collected at several time points throughout the study to measure immune responses; and then 12 weeks after AAV9 inoculation, all animals were euthanized and necropsied to measure immune responses and evaluate GFP expression in tissues.
(EPS)

S2 Fig Expression profiles of 23 cytokines in CSF and plasma of cynomolgus macaques in the two experimental groups, AAV9/GFP controls (G) and AAV9/GFP and rapamycin (GR). Polynomial regressions were applied to the cytokine responses over time and only statistically significant terms retained. In the majority of cases, 83% (76/92), no time dynamics were detectable. Of the remainder, cytokine expression showed mainly linear increases. However, for IL5 in CSF (GR), expression is a quadratic function.
(EPS)

S3 Fig Kinetics of GFP-specific T cell responses altered after treatment with rapamycin. GFP-specific CD4 and CD8 T cell responses were measured at time of gene transfer (D0) and at 28 and 84 days after gene transfer. PBMC were analyzed for the expression of five CD8 effector functions and four CD4 functions by ICS and flow cytometry following in vitro stimulation with GFP-specific peptide pools. Panels A and B depict GFP-specific T cells responses in two treatment groups: AAV9/GFP (controls) and AAV9/GFP + rapamycin. Panels C and D depict AAV9-specific T cell responses in the same groups. Shown: CD8+IFNg+ (#1, 10, 19), CD8+IL2+(#2, 11, 20), CD8+Ki67+(#3, 12, 21), CD8+TNFa+ (#4, 13, 22), CD8+CD107+ (#5, 14, 23), CD4+IFNg+ (#6, 15, 24), CD4+IL2+ (#7, 16, 25), CD4+Ki67+ (#8, 17, 26), CD4+TNFa+ (#9, 18, 27).
(EPS)

S4 Fig Cumulative GFP-specific and AAV9-specific T cell responses. Overall GFP and AAV9-specific T cell responses were determined by calculating the cumulative frequency of CD4+ or CD8+ T cells expressing IFN-**γ**, IL-2, Ki67 or TNF-**α** following in vitro stimulation with overlapping GFP or AAV9 peptide pools. Differences in mean responses between pairs were determined by Mann-Whitney U test. P < 0.05 is considered significant.
(EPS)

S5 Fig Microglial activation in response to AAV9/GFP. Shown are representative 5 micron lumbar spinal cord sections of all study macaques, stained for Iba1. Magnified insets are provided to show areas of positive staining. Macaque ID numbers are provided in each panel. Scale bars present in the control (uninjected) animal are to scale for all images.
(PDF)

S1 Table Intrathecal delivery of AAV9 with a GFP or IL10 transgene in NHP is safe. (DOCX)

S2 Table Rapamycin modulates the kinetics and magnitude of antibody responses to GFP and the AAV9 capsid. (DOCX)

## References

[1] Johnson-Kerner BL, Roth L, Greene JP, Wichterle H, Sproule DM. Giant axonal neuropathy: An updated perspective on its pathology and pathogenesis. Muscle Nerve. 2014;50(4):467–76. doi: 10.1002/mus.24321 . 24947478 10.1002/mus.24321

[2] Demir E, Bomont P, Erdem S, Cavalier L, Demirci M, Kose G, et al Giant axonal neuropathy: clinical and genetic study in six cases. J Neurol Neurosurg Psychiatry. 2005;76(6):825–32. doi: 10.1136/jnnp.2003.035162 . 15897506 10.1136/jnnp.2003.035162 PMC1739689

[3] Pena SD, Opas M, Turksen K, Kalnins VI, Carpenter S. Immunocytochemical studies of intermediate filament aggregates and their relationship to microtubules in cultured skin fibroblasts from patients with giant axonal neuropathy. Eur J Cell Biol. 1983;31(2):227–34. . 6315439

[4] Mussche S, Devreese B, Nagabhushan Kalburgi S, Bachaboina L, Fox JC, Shih HJ, et al Restoration of cytoskeleton homeostasis after gigaxonin gene transfer for giant axonal neuropathy. Hum Gene Ther. 2013;24(2):209–19. doi: 10.1089/hum.2012.107 . 23316953 10.1089/hum.2012.107

[5] Johnson-Kerner BL, Ahmad FS, Diaz AG, Greene JP, Gray SJ, Samulski RJ, et al Intermediate filament protein accumulation in motor neurons derived from giant axonal neuropathy iPSCs rescued by restoration of gigaxonin. Hum Mol Genet. 2015;24(5):1420–31. doi: 10.1093/hmg/ddu556 . 25398950 10.1093/hmg/ddu556 PMC4402342

[6] Medawar PB. Immunity to homologous grafted skin; the fate of skin homografts transplanted to the brain, to subcutaneous tissue, and to the anterior chamber of the eye. Br J Exp Pathol. 1948;29(1):58–69. Epub 1948/02/01. . 18865105 PMC2073079

[7] Zlokovic BV. The blood-brain barrier in health and chronic neurodegenerative disorders. Neuron. 2008;57(2):178–201. Epub 2008/01/25. doi: 10.1016/j.neuron.2008.01.003 . 18215617 10.1016/j.neuron.2008.01.003

[8] Mingozzi F, High KA. Overcoming the Host Immune Response to Adeno-Associated Virus Gene Delivery Vectors: The Race Between Clearance, Tolerance, Neutralization, and Escape. Annu Rev Virol. 2017;4(1):511–34. Epub 2017/09/30. doi: 10.1146/annurev-virology-101416-041936 . 28961410 10.1146/annurev-virology-101416-041936

[9] Tardieu M, Zerah M, Gougeon ML, Ausseil J, de Bournonville S, Husson B, et al Intracerebral gene therapy in children with mucopolysaccharidosis type IIIB syndrome: an uncontrolled phase 1/2 clinical trial. Lancet Neurol. 2017;16(9):712–20. Epub 2017/07/18. doi: 10.1016/S1474-4422(17)30169-2 . 28713035 10.1016/S1474-4422(17)30169-2

[10] Doerfler PA, Nayak S, Corti M, Morel L, Herzog RW, Byrne BJ. Targeted approaches to induce immune tolerance for Pompe disease therapy. Mol Ther Methods Clin Dev. 2016;3:15053 Epub 2016/02/10. doi: 10.1038/mtm.2015.53 . 26858964 10.1038/mtm.2015.53 PMC4729315

[11] Doerfler PA, Todd AG, Clement N, Falk DJ, Nayak S, Herzog RW, et al Copackaged AAV9 Vectors Promote Simultaneous Immune Tolerance and Phenotypic Correction of Pompe Disease. Hum Gene Ther. 2016;27(1):43–59. Epub 2015/11/26. doi: 10.1089/hum.2015.103 . 26603344 10.1089/hum.2015.103 PMC4741206

[12] Kang JJ, Liu IY, Wang MB, Srivatsan ES. A review of gigaxonin mutations in giant axonal neuropathy (GAN) and cancer. Hum Genet. 2016;135(7):675–84. doi: 10.1007/s00439-016-1659-5 . 27023907 10.1007/s00439-016-1659-5

[13] Kantor B, Bailey RM, Wimberly K, Kalburgi SN, Gray SJ. Methods for gene transfer to the central nervous system. Adv Genet. 2014;87:125–97. doi: 10.1016/B978-0-12-800149-3.00003-2 . 25311922 10.1016/B978-0-12-800149-3.00003-2 PMC4519829

[14] Gray SJ, Nagabhushan Kalburgi S, McCown TJ, Jude Samulski R. Global CNS gene delivery and evasion of anti-AAV-neutralizing antibodies by intrathecal AAV administration in non-human primates. Gene Ther. 2013;20(4):450–9. Epub 2013/01/11. doi: 10.1038/gt.2012.101 . 23303281 10.1038/gt.2012.101 PMC3618620

[15] Hinderer C, Bell P, Vite CH, Louboutin JP, Grant R, Bote E, et al Widespread gene transfer in the central nervous system of cynomolgus macaques following delivery of AAV9 into the cisterna magna. Mol Ther Methods Clin Dev. 2014;1:14051 Epub 2014/01/01. doi: 10.1038/mtm.2014.51 . 26052519 10.1038/mtm.2014.51 PMC4448732

[16] Samaranch L, Sebastian WS, Kells AP, Salegio EA, Heller G, Bringas JR, et al AAV9-mediated expression of a non-self protein in nonhuman primate central nervous system triggers widespread neuroinflammation driven by antigen-presenting cell transduction. Mol Ther. 2014;22(2):329–37. Epub 2014/01/15. doi: 10.1038/mt.2013.266 . 24419081 10.1038/mt.2013.266 PMC3918916

[17] Rajendran L, Bali J, Barr MM, Court FA, Kramer-Albers EM, Picou F, et al Emerging roles of extracellular vesicles in the nervous system. J Neurosci. 2014;34(46):15482–9. Epub 2014/11/14. doi: 10.1523/JNEUROSCI.3258-14.2014 . 25392515 10.1523/JNEUROSCI.3258-14.2014 PMC4228143

[18] Powell JD, Pollizzi KN, Heikamp EB, Horton MR. Regulation of immune responses by mTOR. Annu Rev Immunol. 2012;30:39–68. doi: 10.1146/annurev-immunol-020711-075024 . 22136167 10.1146/annurev-immunol-020711-075024 PMC3616892

[19] Garcia JM, Stillings SA, Leclerc JL, Phillips H, Edwards NJ, Robicsek SA, et al Role of Interleukin-10 in Acute Brain Injuries. Front Neurol. 2017;8:244 doi: 10.3389/fneur.2017.00244 . 28659854 10.3389/fneur.2017.00244 PMC5466968

[20] Moore KW, de Waal Malefyt R, Coffman RL, O’Garra A. Interleukin-10 and the interleukin-10 receptor. Annu Rev Immunol. 2001;19:683–765. doi: 10.1146/annurev.immunol.19.1.683 . 11244051 10.1146/annurev.immunol.19.1.683

[21] Spulber S, Bartfai T, Schultzberg M. IL-1/IL-1ra balance in the brain revisited—evidence from transgenic mouse models. Brain Behav Immun. 2009;23(5):573–9. doi: 10.1016/j.bbi.2009.02.015 . 19258032 10.1016/j.bbi.2009.02.015

[22] Sochocka M, Diniz BS, Leszek J. Inflammatory Response in the CNS: Friend or Foe? Mol Neurobiol. 2016 doi: 10.1007/s12035-016-0297-1 . 27889895 10.1007/s12035-016-0297-1 PMC5684251

[23] Schlom J, Jochems C, Gulley JL, Huang J. The role of soluble CD40L in immunosuppression. Oncoimmunology. 2013;2(1):e22546 doi: 10.4161/onci.22546 . 23483661 10.4161/onci.22546 PMC3583923

[24] Sun L, He C, Nair L, Yeung J, Egwuagu CE. Interleukin 12 (IL-12) family cytokines: Role in immune pathogenesis and treatment of CNS autoimmune disease. Cytokine. 2015;75(2):249–55. doi: 10.1016/j.cyto.2015.01.030 . 25796985 10.1016/j.cyto.2015.01.030 PMC4553122

[25] Araki K, Ellebedy AH, Ahmed R. TOR in the immune system. Curr Opin Cell Biol. 2011;23(6):707–15. Epub 2011/09/20. doi: 10.1016/j.ceb.2011.08.006 . 21925855 10.1016/j.ceb.2011.08.006 PMC3241972

[26] Malek TR, Bayer AL. Tolerance, not immunity, crucially depends on IL-2. Nat Rev Immunol. 2004;4(9):665–74. doi: 10.1038/nri1435 . 15343366 10.1038/nri1435

[27] Takatsu K. Interleukin-5 and IL-5 receptor in health and diseases. Proc Jpn Acad Ser B Phys Biol Sci. 2011;87(8):463–85. doi: 10.2183/pjab.87.463 . 21986312 10.2183/pjab.87.463 PMC3313690

[28] Jabri B, Abadie V. IL-15 functions as a danger signal to regulate tissue-resident T cells and tissue destruction. Nat Rev Immunol. 2015;15(12):771–83. doi: 10.1038/nri3919 . 26567920 10.1038/nri3919 PMC5079184

[29] Soehnlein O, Steffens S, Hidalgo A, Weber C. Neutrophils as protagonists and targets in chronic inflammation. Nat Rev Immunol. 2017;17(4):248–61. doi: 10.1038/nri.2017.10 . 28287106 10.1038/nri.2017.10

[30] Tornoe J, Kusk P, Johansen TE, Jensen PR. Generation of a synthetic mammalian promoter library by modification of sequences spacing transcription factor binding sites. Gene. 2002;297(1–2):21–32. . 12384282 10.1016/s0378-1119(02)00878-8

[31] Grieger JC, Soltys SM, Samulski RJ. Production of Recombinant Adeno-associated Virus Vectors Using Suspension HEK293 Cells and Continuous Harvest of Vector From the Culture Media for GMP FIX and FLT1 Clinical Vector. Mol Ther. 2016;24(2):287–97. doi: 10.1038/mt.2015.187 . 26437810 10.1038/mt.2015.187 PMC4817810

