# Correction: Sustained AAV9-mediated expression of a non-self protein in the CNS of non-human primates after immunomodulation

**DOI:** 10.1371/journal.pone.0207077

**Published:** 2018-11-01

**Authors:** Arlene I. Ramsingh, Steven J. Gray, Andrew Reilly, Michael Koday, Debbie Bratt, Merika Treants Koday, Paul Munson, Robert Murnane, Jeremy Smedley, Yuhui Hu, Anne Messer, Deborah Heydenburg Fuller

Dr. Paul Munson should be included in the author byline. He should be listed as the seventh author, and his affiliation is 6: the Department of Microbiology, University of Washington, Seattle, Washington, United States of America. The contributions of the author are as follows: Data curation, formal analysis, methodology, and validation.
