# Supplementary figures and images for: Sustained AAV9-mediated expression of a non-self protein in the CNS of non-human primates after immunomodulation
Source: PLoS One. 2018 Jun 6;13(6):e0198154. doi: 10.1371/journal.pone.0198154 (PMC5991358; doi:10.1371/journal.pone.0198154)

S5 Figure

GFP only

GFP + rapamycin

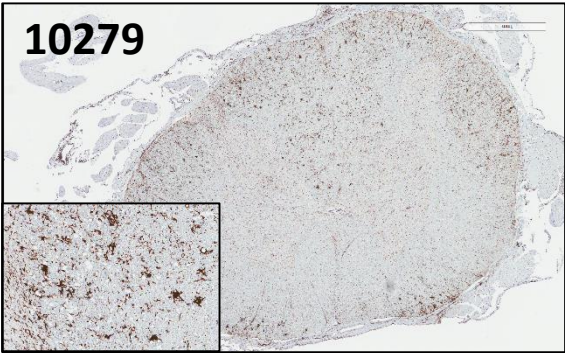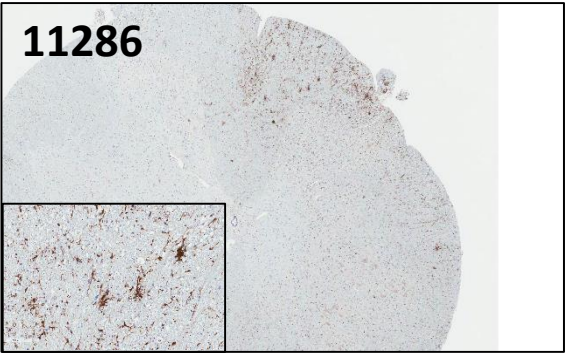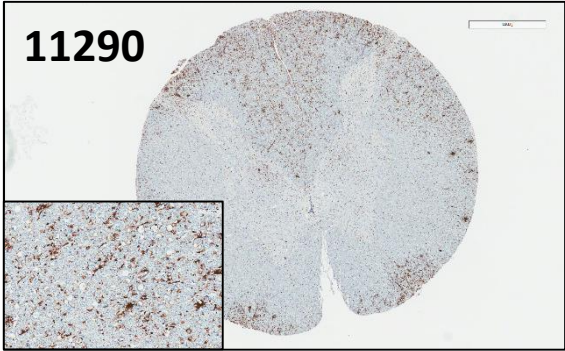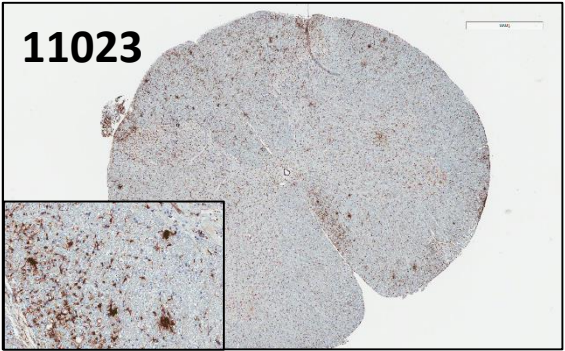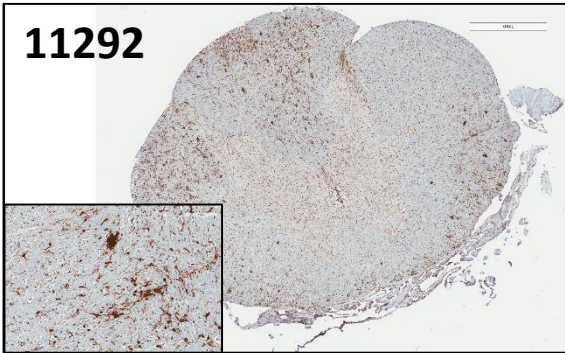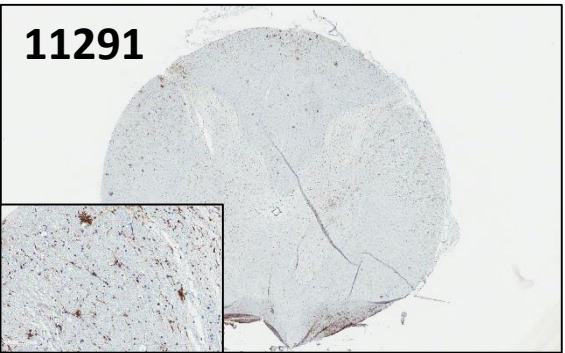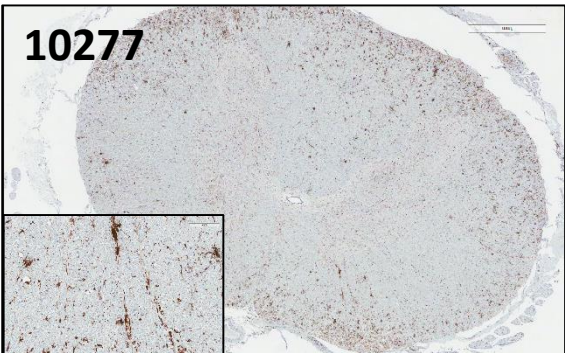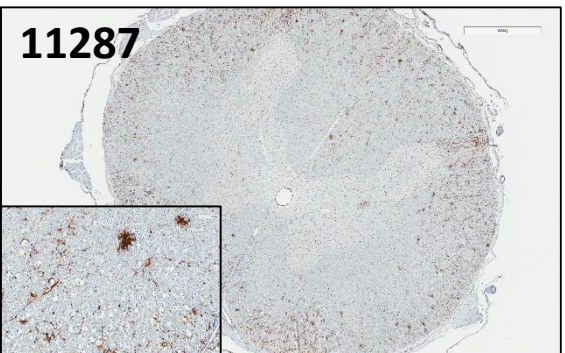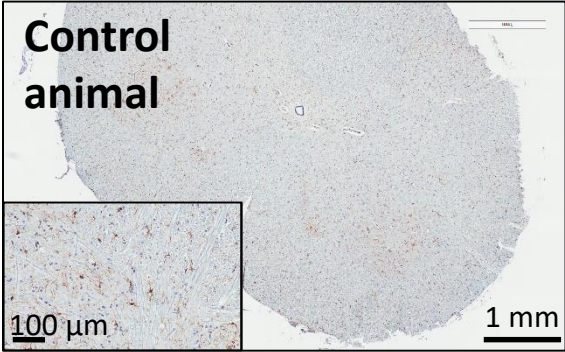

Supplement: S5 Fig — Shown are representative 5 micron lumbar spinal cord sections of all study macaques, stained for Iba1. Magnified insets are provided to show areas of positive staining. Macaque ID numbers are provided in each panel. Scale bars present in the control (uninjected) animal are to scale for all images. (PDF) [file pone.0198154.s005.pdf]
