# Supplementary material for: Sustained AAV9-mediated expression of a non-self protein in the CNS of non-human primates after immunomodulation
Source: PLoS One. 2018 Jun 6;13(6):e0198154. doi: 10.1371/journal.pone.0198154 (PMC5991358; doi:10.1371/journal.pone.0198154)
Supplement: S1 Table — (DOCX) [file pone.0198154.s006.docx]

**Table S1**

| **Treatment group** | **Overall Health** | **Activity** | **Motor function** | **Complete Blood Count (CBC)** |
| --- | --- | --- | --- | --- |
| AAV9/GFP  (control) | No adverse  effects | No adverse  effects | No adverse  effects | Values unremarkable; Slight eosinophilia; Mild leukocytosis; Slight monocytosis |
| AAV9/GFP plus  Rapamycin | No adverse  Effects | No adverse  Effects | No adverse  effects | Values unremarkable; Mild leukocytosis (possible stress) |
