# Supplementary material for: Sustained AAV9-mediated expression of a non-self protein in the CNS of non-human primates after immunomodulation
Source: PLoS One. 2018 Jun 6;13(6):e0198154. doi: 10.1371/journal.pone.0198154 (PMC5991358; doi:10.1371/journal.pone.0198154)
Supplement: S2 Table — (DOCX) [file pone.0198154.s007.docx]

**Table S2**

| **Parameter** | **Treatment** | |
| --- | --- | --- |
|  | AAV9/GFP | AAV9/GFP and rapamycin |
| Time (days) to plasma AAV9 Ab | 5-19 | 25-48  (delayed) |
| Time (days) to CSF AAV9 Ab | 1.5-51 | 18-51 |
| Time (days) to plasma GFP Ab | 10-27 | 26-39  (delayed) |
| Time (days) to CSF GFP Ab | 18-41 | 9-29  (faster) |
| Maximal plasma AAV9 Ab | ^1^Moderate | ^2^Reduced (in 3 of 4 animals) |
| Maximal CSF GFP Ab | ^3^High | ^4^Reduced (in 3 of 4 animals) |

OD450 nm: ^1^Moderate, 1.042-2.330; ^2^Reduced, 0.486-1.810;

^3^High 3.738-4.0; ^4^Reduced, 0.25-3.845.
